# 10^−7^ M genistein partially alleviates 10^−7^ M MEHP unfavorable effects in a new modified fetal rat testis culture system

**DOI:** 10.3389/fcell.2022.987928

**Published:** 2022-08-29

**Authors:** Tong-Dian Zhang, Yu-Bo Ma, Ming Gao, He-Cheng Li, Zi-Ming Wang, Tie Chong, Lian-Dong Zhang

**Affiliations:** ^1^ Department of Urology, The Second Affiliated Hospital of Xi’an Jiaotong University, Xi’an, Shaanxi, China; ^2^ Department of Andrology, Liaocheng People’s Hospital, Liaocheng, Shandong, China; ^3^ Department of Nephrology, Xi’an No. 4 Hospital, Xi’an, Shaanxi, China

**Keywords:** mono-(2-ethylhexyl) phthalate, genistein, fetal testis, organ culture, oxidative stress

## Abstract

**Background:** Recent studies revealed that some common endocrine-disrupting chemicals (EDCs) including phthalates and phytoestrogens may exhibit low-dose effects properties. However, how low dose of these EDCs and their mixture would affect fetal rat testis development still needs further investigation. Moreover, testis organ culture system also needs further modification to provide an effective tool for *ex vivo* EDCs study.

**Methods:** We firstly modified the agarose organ culture system, in which fetal rat testes were cultured for 4 days (d1 to d4) on agarose gels held by Millicell inserts. Then we used the modified agarose culture system to study the combined effects of multiple EDCs exposure. 15.5 dpc fetal rat testes were isolated and treated with vehicle, MEHP (0.1 μmol/L), GEN (0.1 μmol/L) or MEHP (0.1 μmol/L) + GEN (0.1 μmol/L). Parameters concerning testicular cell development and function were evaluated, trying to gain insight into the early molecular events after multiple EDCs exposure.

**Results:** The development of somatic, germ cells and seminiferous tubule in 15.5 dpc fetal rat testis was better sustained in the modified agarose culture system. Based on the modified system, we found that MEHP at 0.1 μmol/L induced alterations in gonocyte markers, antioxidative enzyme activity as well as transient reduction of testosterone production, accompanied by mitochondria swelling in gonocytes and Sertoli cells. No obvious morphological and histological alterations were observed in all treated groups. However, coadministration of genistein at 0.1 μmol/L partially alleviated MEHP-induced fetal testis damage *ex vivo* through enhancement of antioxidative action. MEHP at low dose still showed weak endocrine disrupting properties but did not exhibit typical low-dose effects.

**Conclusion:** Our findings indicated that the modified agarose culture system could better mimic testicular microenvironment without obvious hypoxic cell damage. Furthermore, low dose of MEHP induced mild disruption to fetal testis development, cotreatment of genistein at low dose attenuated MEHP induced fetal testis injuries in part by balancing redox state, indicating that low dose of genistein may partially protect fetal testis from phthalates induced injury.

## 1 Introduction

Over the last two decades, endocrine disrupting chemicals (EDCs)-related male reproductive system disorder has aroused growing concerns (Panner Selvam and Sikka, 2022). It was found that certain EDCs exhibited adverse effects on male reproductive system development and function by interfering with natural hormones production and metabolism in the body (Nassouri et al., 2012). Although extensive studies were done to make clear the reproductive effects following single EDC exposure, however, knowledge gaps still exist on fetal testis development after multiple EDCs exposure at low doses.

It was found that phthalates and phytoestrogens, two kinds of the most pervasive EDCs, were widely detected in human adults and infants (Jefferson and Williams, 2011; Lin et al., 2011). Our previous studies demonstrated that *in utero* coexposure to genistein (GEN) and di (2-ethylhexyl) phthalate (DEHP) altered fetal testis development in a way different from single chemical exposure (Zhang et al., 2013; Jones et al., 2015). Genistein, one of the most prevalent phytoestrogen in soy products (Zhang et al., 2018), exhibited weak estrogenic and antioxidative properties (Zhao et al., 2016; Sarkar et al., 2022). It is important to note that genistein exhibited biphasic effects on cell proliferation in breast cancer cell lines at different concentrations (Şöhretoğlu et al., 2021). Other studies revealed that soy isoflavones can alleviate EDCs induced oxidative injuries by modulating antioxidative system (Zhang et al., 2018; Fan et al., 2022). DEHP and its bioactive metabolite mono-(2-ethylhexyl) phthalate (MEHP) are primarily used as the chemical additives of polyvinyl chloride plastics (Zhang et al., 2017). Culty M (13) found that DEHP and MEHP activated peroxisome proliferator-activated receptors (PPARs) and disrupted fetal testosterone biosynthesis. It was also found that MEHP elevated reactive oxygen species (ROS) level in MA-10 Leydig cells and impaired mitochondrial function which may finally lead to the cellular redox state imbalance (Zhou et al., 2019).

Prenatal reproductive system is confirmed to be critically sensitive to environmental chemicals. During this stage, fetal Sertoli cells and Leydig cells play fundamental roles in generating hormones required for germ cells differentiation and proliferation. Testosterone was synthesized by fetal Leydig cells around 15.5 days postcoitum (dpc) in the rat and reached a peak at 18.5 dpc (Habert and Picon, 1984). Further studies revealed that the critical window of EDCs exposure for fetal testis was between gestational day 16–18 (Carruthers and Foster, 2005). However, the lack of efficient detoxifying system and blood-testis barrier makes fetal testis especially sensitive to EDCs (Jones et al., 2015), finally leading to the disruption of early primordial germ cell determination and gonadal differentiation (Aksglaede et al., 2006; Perobelli, 2014).

Previous studies basically evaluated single EDC induced reproductive system alteration *in vitro*, *ex vivo*, and *in vivo* (Basak et al., 2020; Horie et al., 2021; Munier et al., 2021), however, the combined effects on fetal testis development after multiple EDCs exposure at low doses were rarely investigated (Walker et al., 2021). Moreover, most *in vivo* studies focused on *in utero* exposure to EDCs, it was not easy to distinguish whether EDCs exert direct and indirect effects on testis development. Our previous study revealed that *in utero* exposure to genistein at low dose alleviate DEHP-induced neonatal testis injuries at (Jones et al., 2015), highlighting the necessity and importance to evaluate the combined effects of EDCs exposure at low doses. Moreover, due to the technical difficulties to obtain and culture fetal testis, *ex vivo* exposure risk evaluation on fetal testis was rarely reported. To overcome those difficulties, multiple efforts have been made to simulate physical process of fetal testis development. Of all approaches, three-dimensional culture systems preserving intact testicular architecture and intercellular communications were optimal alternatives for fetal testis studies (Boulogne et al., 2003), and organ culture system was regarded as one of the most powerful *ex vivo* methods. In the early models, the testis was put on a grid coated with agar to study the regulation of the testosterone production (Habert et al., 2001) and spermatogenesis in the neonatal testis (Schlatt et al., 1999), however, the cultured testes usually exhibited poor viability and apparent necrosis. The floating filter system using Millipore culture filter was later developed by Habert and Brignaschi (1991) and showed superiority of less necrosis and stronger steroidogenic activity over grid system. However, the culture period usually lasted less than 3 days and necrosis was still inevitable, especially for fetal testis culture.

In this study, we firstly modified the fetal testis organ culture system and then used the modified system to study the combined effects of genistein and MEHP at even low dose. Parameters related to testicular development and function were evaluated, trying to explore an effective *ex vivo* model and hoping to understand the molecular events following fetal testis exposure to multiple EDCs.

## 2 Materials and methods

### 2.1 Animal feeding and sample collection

Sexually mature SPF Sprague-Dawley rats were supplied by the Experimental Animal Center of Xi’an Jiaotong University and kept with 12:12-h light: dark cycle with *ad libitum* access to water and food. Every two female Sprague-Dawley rats were mated with one male rat overnight and vaginal smears were examined. The day of sperm detection was recorded as 0.5 dpc. Before the initiation of this study, ethical clearance was obtained from Committee on Animal Research and Ethics of Xi’an Jiaotong University (Xi’an, China).

On 15.5 dpc, pregnant rats were anesthetized and the fetuses were dissected aseptically under anatomical microscope. Fetal testes were dissected and immersed in the cold culture medium. For rat fetuses older than 13.5 dpc, the fetal testes can be identified morphologically based on the characteristic testicular vessels (Livera et al., 2006).

### 2.2 Comparison of different organ culture systems

The fetal testes were harvested on 15.5 dpc and were divided into control group and modified group. In the control, Millicell insert (PICM-01250, Millipore Corp., United States) was used as stand for fetal testis culture. In the modified group, 1.5% (w/v) agarose gel cube (10 mm^3^ × 5 mm^3^ × 5 mm^3^) set in the Millicell was used as stand for fetal testis culture. Briefly, each harvested fetal rat testis was put on one Millicell insert in the control, and each agarose gel cube in the modified group was loaded with one fetal testis. Then they were transfered into the 24-well plates containing 600 μl culture medium. The fetal testes were cultured for 4 days at 37°C in humidified atmosphere of 5% CO2 and 95% air. It was counted as day 0 (d0) when starting organ culture and the next 4 days were counted as d1, d2, d3, d4.

The culture medium we used was Dulbecco’s Modified Eagle’s Medium (DMEM) mixed 1:1 with Ham’s F12 (DMEM/F12) medium (TransGen Biotech, Beijing, China) supplemented with 10% (v/v) KnockoutTM Serum Replacement (KSR, Gibco, United States), penicillin (100 IU/ml) and streptomycin (100 IU/ml) (TransGen Biotech, Beijing, China). Testis morphology, histology, and testosterone production were evaluated.

### 2.3 Chemicals and 15.5 dpc testis treatment

After testing the culture efficacy of different models, we applied the modified agarose culture system to evaluate reproductive effects of MEHP and genistein.

MEHP(CAS:4376-20-9, Accustandard Inc., Connecticut, United States) and genistein (CAS: 446-72-0, Sigma-Aldrich Inc., St. Louis, United States) were obtained and dissolved in dimethylsulfoxid. The cultured fetal testes were treated with vehicle (Control), GEN (0.1 μmol/L, G), MEHP (0.1 μmol/L; M), or GEN (0.1 μmol/L) + MEHP (0.1 μmol/L) respectively for 4 days. Every four fetuses from the same mother rats were divided into four groups and six fetal rats were included in each group, trying to minimize differences between groups and keep better homogeneity.

### 2.4 RNA extraction and quantitative real-time PCR

The detailed procedure of RNA extraction was based on the protocol of EasyPure RNA Kit (Transgen Biotech, China), and RevertAid™ First Strand cDNA Synthesis Kit (Thermo Fisher Scientific, United States) was used to synthesize cDNA. Real-time quantitative PCR was done using TB Green Premix Ex Taq II (Takara, Japan) with total reaction volume of 20 μl on the Bio-Rad CFX Connect Real-Time PCR Detection System (Bio-Rad, United States). Gapdh was set as an internal control. The amplification process was initiated with a denaturation cycle at 95°C for 2 min, followed by 39 cycles of 95°C for 10 s and 60°C for 30 s. All analyses were performed in triplicate samples. Quantitative analysis of gene expression was evaluated using the 2^−∆Ct^ algorithm. The gene names and primer sequences are listed in Table 1.

**TABLE 1:** The genes and primer sequences.

Gene name	Accession no.	Forward primer	Reverse primer
Gapdh	NM_017008.4	5-TGG​GTG​TGA​ACC​ACG​AGA​A-3	5-GGC​ATG​GAC​TGT​GGT​CAT​GA-3
Nrf2	NM_031789.2	5-ACG​GTG​GAG​TTC​AAT​GAC-3	5-TGT​TGG​CTG​TGC​TTT​AGG-3
Pdgfrα	NM_012802.1	5-GCT​ACA​CGT​TTG​AGC​TGT​CAA​C-3	5-ATG​GTG​GTC​ATC​CAC​AAG​C-3
Cyp11a1	NM_017286.2	5-CAC​GCA​CTT​CCG​GTA​CTT​GG-3	5-CGG​ATA​TTT​CCA​GCT​CTG​CAA​TCC​G-3
Tspo	NM_012515.1	5-CGC​AAT​GGG​AGC​CTA​CTT​TGT​GCG-3	5-GCC​AGG​AGG​GTT​TCT​GCA​AG-3
Hsd3β	NM_001007719.3	5-GAC​CAG​AAA​CCA​AGG​AGG​AA-3	5-CTG​GCA​CGC​TCT​CCT​CAG-3
Amh	NM_012902.1	5-CGG​GCT​GTT​TGG​CTC​TGA​TTC​CCG-3	5-GTG​GGT​GGC​AGC​AGC​ACT​AGG-3
Wt-1	NM_031534	5-CGG​TCG​TCT​TCA​GGT​GGT​CGG​ACC​G-3	5-GCA​CCA​AAG​GAG​ACA​CAC​AGG​T-3
Pdgfrβ	NM_031525.1	5-ATG​GAC​ATG​AGC​AAG​GAT​GA-3	5-GTC​CGC​GTA​TTT​GAT​GTG​TC-3
Dazl	NM_001109414.1	5-TGA​AGT​TGA​TCC​AGG​AGC​TG-3	5-CCA​CTG​TCT​GTA​TGC​TTC​GG-3
Sod1	NM_017050.1	5-AGA​GAG​GCA​TGT​TGG​AGA​CC-3	5-TAG​TAC​GGC​CAA​TGA​TGG​AA-3
Sod2	NM_017051.2	5-GGC​TTG​GCT​TCA​ATA​AGG​AG-3	5-TAG​TAA​GCG​TGC​TCC​CAC​AC-3
Cat	NM_012520.1	5-TTC​ATC​AGG​GAT​GCC​ATG​T-3	5-GGG​TCC​TTC​AGG​TGA​GTT​TG-3
Hsp90α	NM_175761.2	5-TTT​CGT​GCG​TGC​TCA​TTC​T-3	5-AAG​GCA​AAG​GTT​TCG​ACC​TC-3
Tgfβ	NM_021578.2	5-CCG​CAA​CAA​CGC​AAT​CTA​TG-3	5-AAG​CCC​TGT​ATT​CCG​TCT​CC-3
Gfrα1	NM_012959.1	5-GTA​CTT​CGC​GCT​GCC​ACT-3	5-GCT​TTC​ACA​CAG​TCC​AGA​CG-3
Sohlh2	NM_001034961.1	5-AGC​CAG​CTC​CAG​TTG​TCT​GT-3	5-GAT​GCT​GGA​TGA​GGC​AGT-3

### 2.5 Testosterone assay

The culture medium was collected every 24 h and stored at −80°C until radioimmunoassay could be performed (Tianjin Nine Tripods Medical &Bioengineering Co., Ltd., Tianjin, China). Each sample was assayed in triplicate and performed by an independent investigator blind to treatment.

### 2.6 Redox state assay

Total antioxidant capacity (T-AOC), superoxide dismutase (SOD) activity, total glutathione (GSH) and malondiadehyde (MDA) (Yao et al., 2015) concentration in culture medium were spectrophotometrically evaluated using the commercial kits (Nanjing Jiancheng Bioengineering Institute, Nanjing, China). The results of T-AOC and MDA were expressed as nmol/ml, SOD activity was expressed as U/ml and GSH was expressed as μmol/ml.

### 2.7 Testicular morphology and histology

The cultured fetal testis development was observed under inverted microscope for 4 days. On d4, the cultured testes were collected and fixed in 4% (w/v) paraformaldehyde and embedded in paraffin. Tissue Sections (5 µm) were dewaxed, rehydrated and stained with hematoxylin and eosin. Pictures were taken with an Olympus microscope.

### 2.8 Transmission electron microscopy

Samples were fixed in 2.5% (w/v) glutaraldehyde in 0.1 M sodium phosphate buffer, then post-fixed in 1% (w/v) osmium tetroxide for 2 h, dehydrated through a series of graded ethanol and embedded in Emix resin. Ultra-thin sections (60 nm) were cut and double-stained with uranyl acetate and lead citrate, and observed under a transmission electron microscope (H-7650, Hitachi, Japan).

### 2.9 Statistical analysis

Data were expressed as the mean ± standard error of the mean and analyzed using SPSS 15.0 (SPSS Inc., Chicago, IL, United States). Statistical analysis was performed using one-way ANOVA with post hoc TURKEY when equal variances were assumed otherwise followed by Games-Howell for comparisons of more than two groups, or unpaired two-tailed *t*-test for comparisons of two groups. A difference with *p* < 0.05 was considered to be statistically significant.

## 3 Results

### 3.1 Comparison of organ culture models

#### 3.1.1 15.5 dpc fetal testis preparation and identification

**FIGURE 1:**
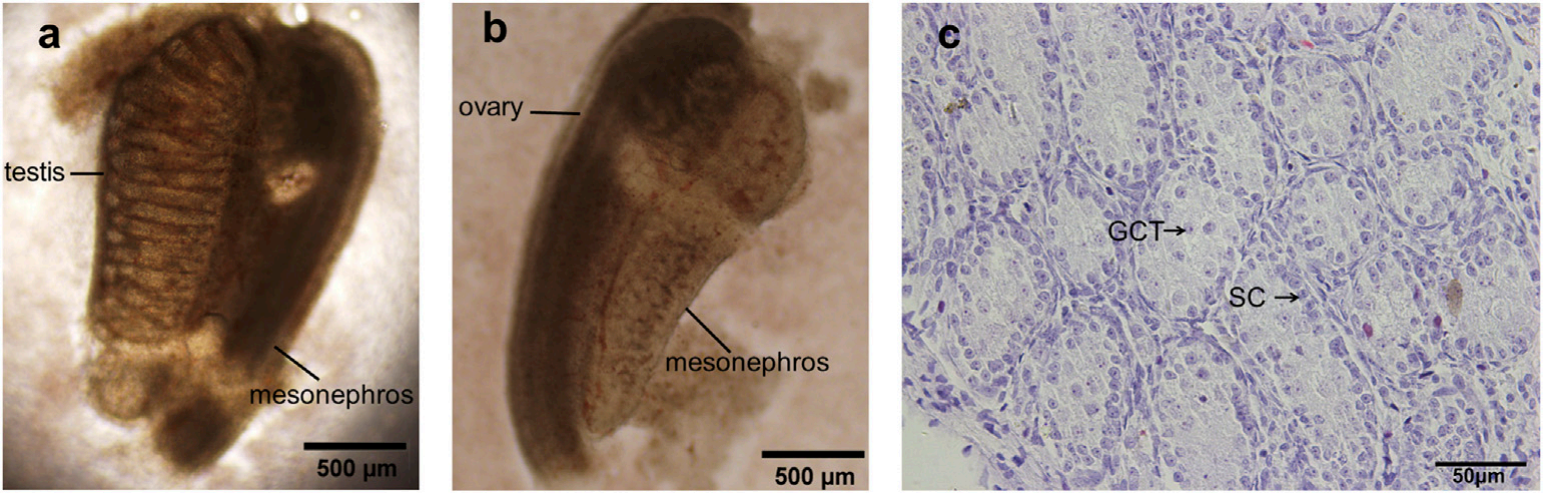
15.5 dpc fetal testis preparation and identification. **(A)** The testis was rounder than ovary and contained a testicular vessel; **(B)** Ovary was relatively longer and spots were visible under anatomic microscope; **(C)** H&E staining of dpc 15.5 fetal testis, intact seminiferous tubules with no lumen were present, gonocytes and Sertoli cells were normally displayed. GCT, gonocytes; SC, Sertoli cells. **(A,B)** ×100 magnification, scale bars indicate 500 μm, **(C)** ×400 magnification, scale bars indicate 50 µm.

15.5dpc female SD rats were anesthetized and uteri were taken out. After removing amnions and placenta, the abdomen of fetuses was then dissected along the midline. The testes were then identified morphologically under anatomic microscope which were rounder than ovaries and contained testicular vessel (Figure 1A), while ovaries were relatively longer and spots were visible under anatomic microscope (Figure 1B).

Fetal testes were further identified by hematoxylin and eosin staining before the initiation of organ culture. In the sections (Figure 1C), intact seminiferous tubules were present and lumen was still not formed, verifying the successful acquisition of dpc 15.5 fetal testis. Typically the gonocytes are large oval or rounded cells with one or two nucleoli, and they occupy the center of lumen-less seminiferous cord, easily distinguishable from adjacent Sertoli cells. By contrast, Sertoli cells locate in the periphery of the lumen-less seminiferous cord, the cells are oval or round but size is smaller than gonocytes, the nuclei of Sertoli cells are stained darker than those of gonocytes.

#### 3.1.2 Microscopic views of the cultured fetal testis

**FIGURE 2:**
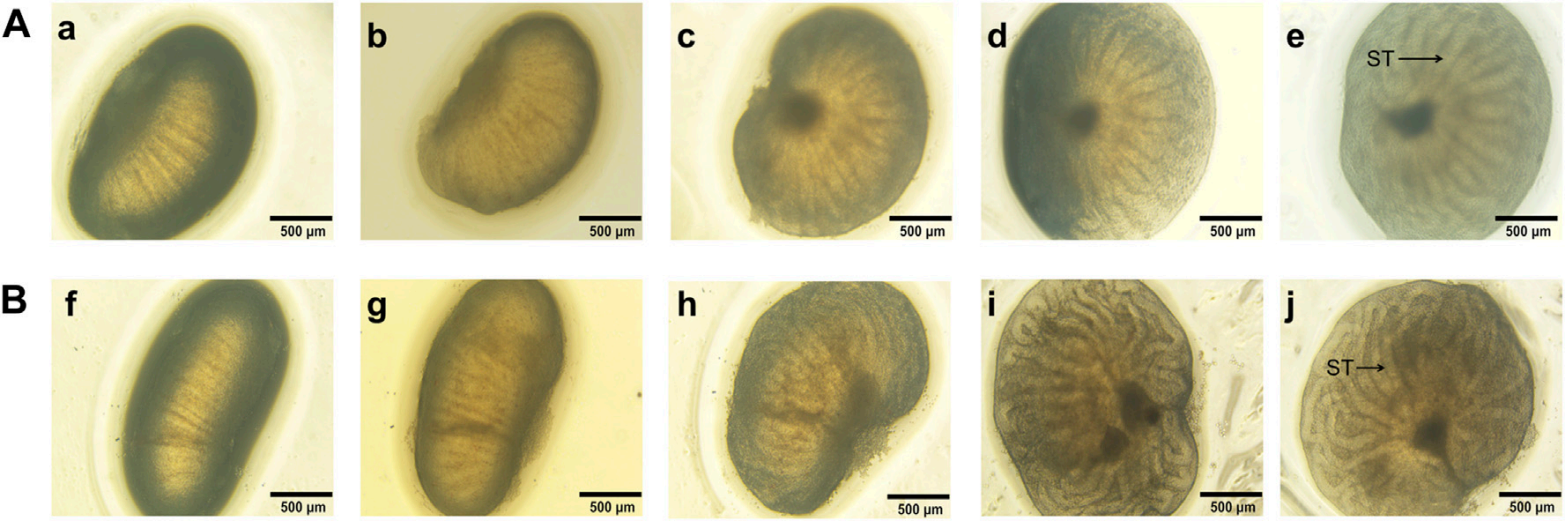
Microscopic views of 15.5 dpc fetal testis cultured for 4 days a, f: d0; b, g: d1; c, h: d2; d, i: d3; e, j: d4. The seminiferous tubules were firstly observed on d2 in the modified group and the length and thickness of seminiferous tubules apparently increased. By contrast, the seminiferous tubules were firstly seen on d4 in control group, the seminiferous tubules were more coiled in modified group compared to control on d4. ST, seminiferous tubules.**(A)**: control group, **(B)**: the modified group. ×40 magnification. Scale bars indicate 500 µm.

The size of the *ex vivo* cultured fetal testes increased during four consecutive days in both control group and the modified group. The seminiferous tubules were firstly observed on d2 in the modified group and the length and thickness of seminiferous tubules showed apparent increase in the following days (Figure 2). The seminiferous tubules were firstly seen on d4 in control group, the seminiferous tubules were more coiled in modified group compared to control on d4, indicating that fetal testis continued to develop *ex vivo* and the modified agarose organ culture system could better mimic physiological process.

#### 3.1.3 Testosterone production by cultured fetal testis *ex vivo*

**FIGURE 3:**
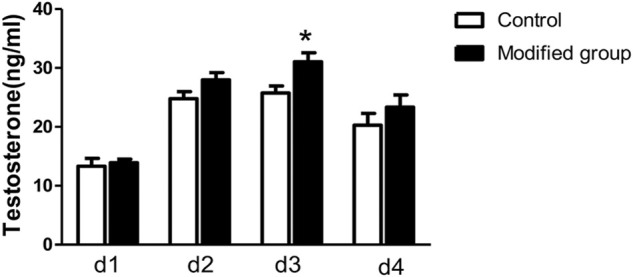
Testosterone production by cultured fetal testis *ex vivo*. *, significantly different from control at *p* < 0.05.

Testosterone concentration of the culture medium was shown in Figure 3. Testosterone production was elevated in the first 3 days and began to decrease on day four in both control and modified groups. Further comparison found that on d3 testosterone concentration of the modified group was significantly higher in comparison with the control group (*p <* 0.05).

#### 3.1.4 Testicular histology

**FIGURE 4:**
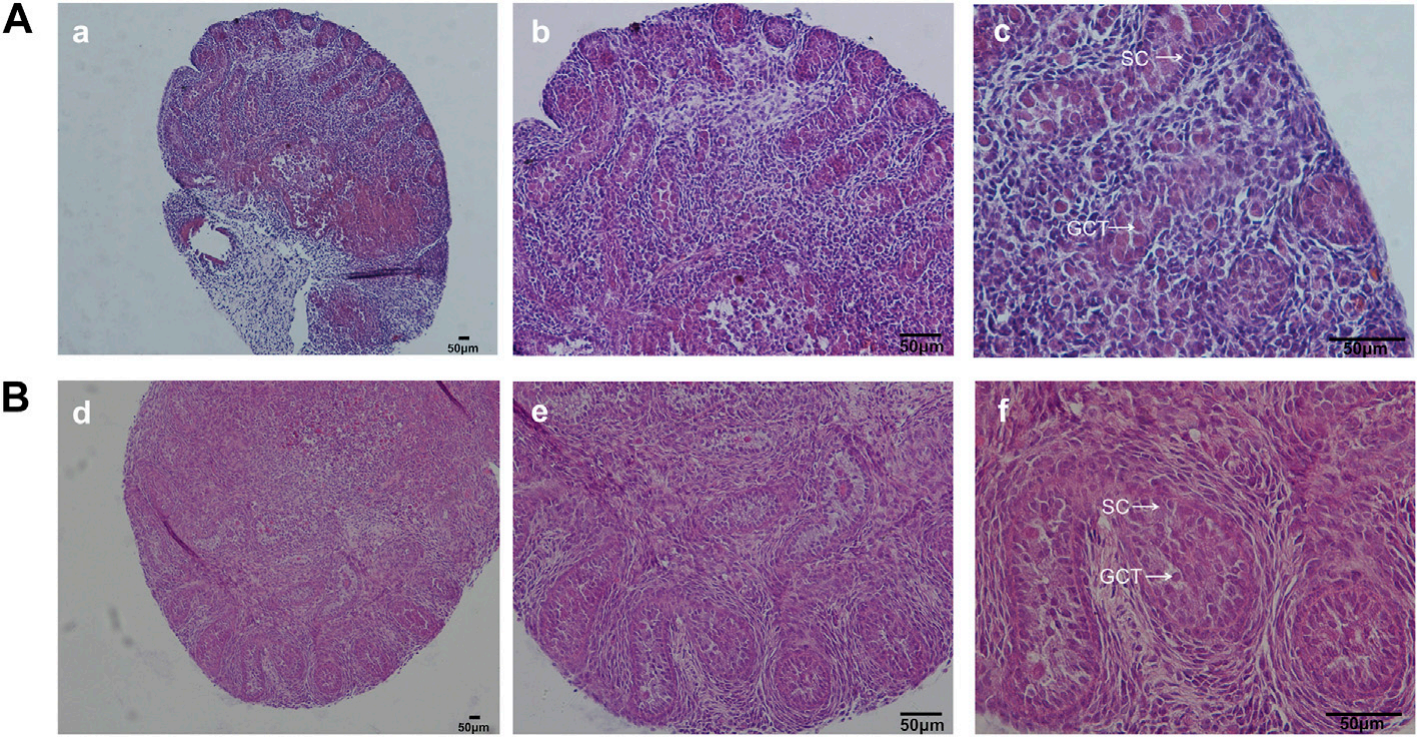
H&E staining of fetal testis on d4. In the modified group, H&E staining showed normal testicular structure. By contrast, testicular sections in the control group exhibited smaller tubule diameter and underdeveloped seminiferous tubules. GCT, gonocytes; SC, Sertoli cells.**(A)**: control group, **(B)**: the modified group. a, d: ×100; b, e: ×200; c, f: ×400 magnification. Scale bars indicate 50 µm.

After cultured for 4 days, intact seminiferous tubule structure was well sustained in the modified group, no obvious lesions including necrosis or tubular vacuole was observed (Figure 4). Gonocytes located centrally in the seminiferous tubules could readily be distinguished from the Sertoli cells. By contrast, testicular sections in the control group exhibited smaller tubule diameter and underdeveloped seminiferous tubules, in accordance with the microscopic findings under inverted microscope, manifesting that fetal testis development was better sustained based on appropriate oxygen and nutrient supply in the modified organ culture system.

### 3.2 Effects of low dose of genistein and mono-(2-ethylhexyl) phthalate on cultured fetal rat testis

#### 3.2.1 Gene expression of germ cell, Sertoli cell, and Leydig cell markers

**FIGURE 5:**
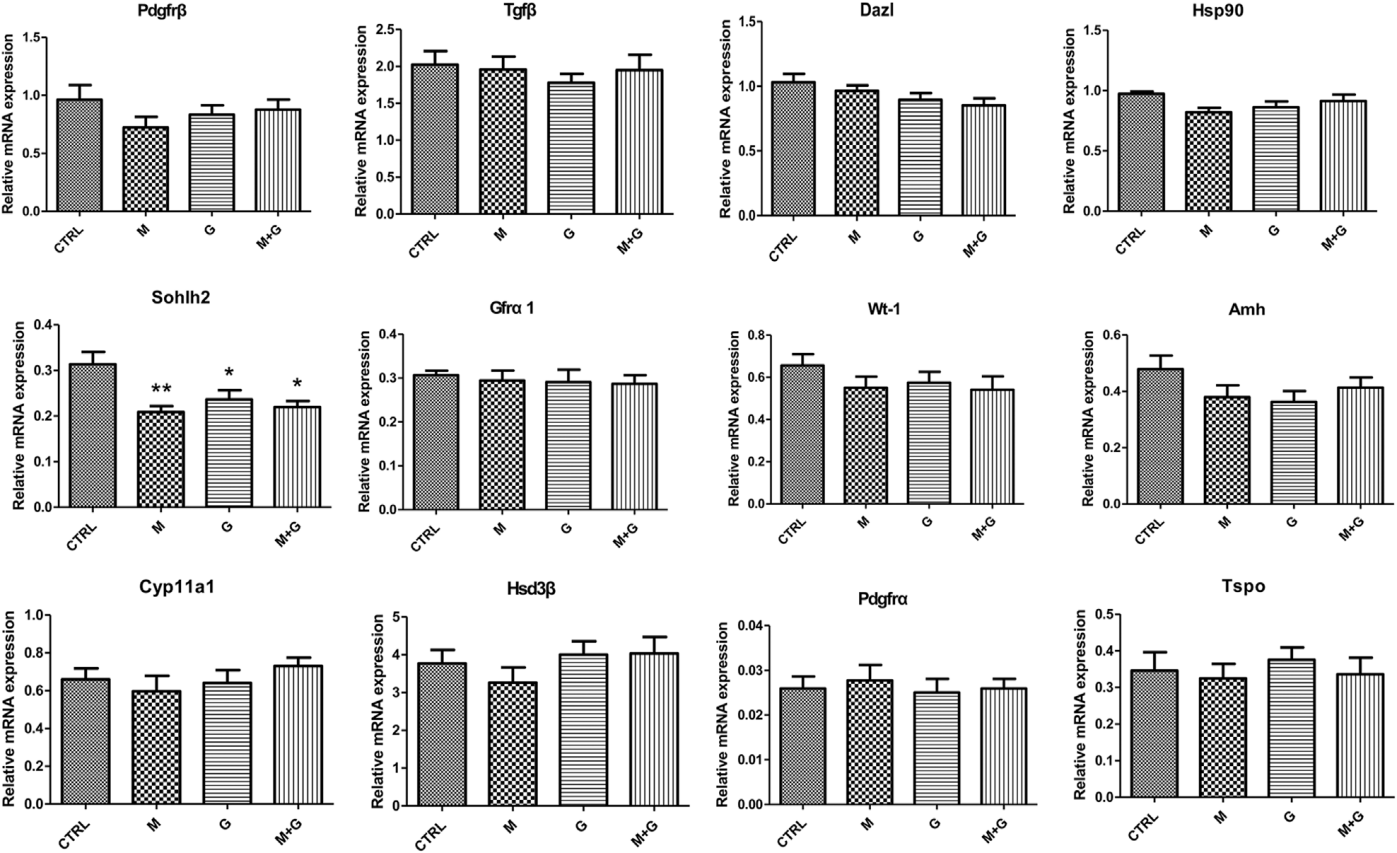
Gene expression of fetal germ cell, Sertoli cell and Leydig cell markers. *, significantly different from CTRL at *p* < 0.05; **, significantly different from CTRL at *p* < 0.01.

Gene expression of germ cell (Pdgfrβ, Tgfβ, Dazl, Hsp90, Sohlh2, and Gfrα1), Sertoli cell (Wt-1 and Amh) and Leydig cell (Cyp11a1, Hsd3β, Tspo, and Pdgfrα) markers is shown in Figure 5. Expression of Pdgfrβ, Tgfβ, Dazl, Hsp90, and Gfrα1 showed no significant alterations between each group (*p >* 0.05). Sohlh2 expression after MEHP and genistein exposure exhibited significant decrease in comparison with control (*p* < 0.01 and 0.05 respectively), and the combined exposure of MEHP and genistein also induced significant decrease compared to control (*p* < 0.05).

No significant alterations was observed in Wt-1 and Amh expression in all groups (*p* > 0.05), indicating that low dose of MEHP, genistein and their mixture exerted no disrupting effects on Sertoli cell differentiation.

For Leydig cell makers, we found that different treatment induced no significant difference in the expression of Cyp11a1, Hsd3β, Tspo, and Pdgfrα (*p* > 0.05), indicating that low dose of MEHP , genistein and their mixture may not aggravate fetal Leydig cell injury.

#### 3.2.2 Gene expression of Nrf2, antioxidative genes, and analysis of medium redox state

**FIGURE 6:**
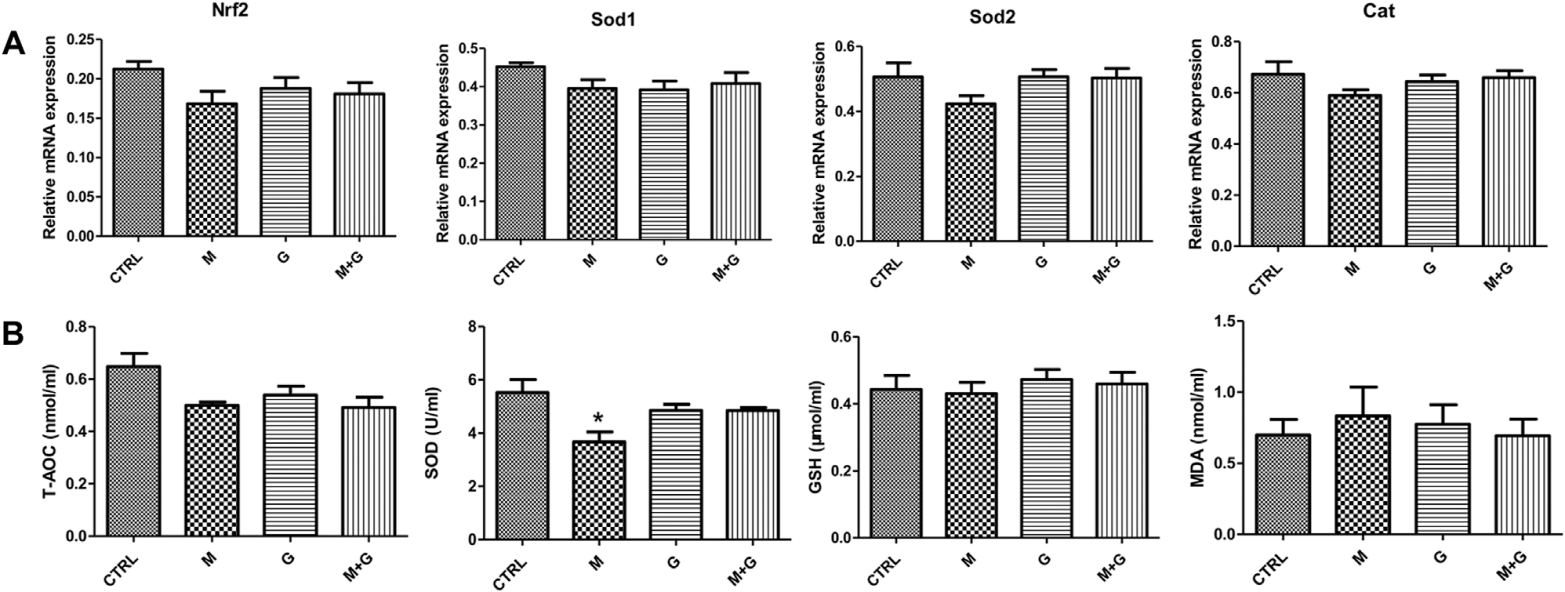
Gene expression of Nrf2, antioxidative genes and analysis of medium redox state. **(A)** Gene expression of Nrf2 and antioxidative genes; **(B)** Analysis of medium redox state. *, significantly different from CTRL at *p* < 0.05.

We further evaluate the gene expression of Nrf2 and downstream antioxidative genes (Figure 6). MEHP, genistein and their combination induced no significant alterations of Nrf2, Sod1, Sod 2, and Cat expression (*p >* 0.05).

In each treated group, T-AOC, GSH and MDA showed no significant alteration compared with control. However, medium SOD activity after MEHP treatment decreased significantly (*p* < 0.05), while the mixture group showed no significant reduction compared with control (*p* > 0.05), indicating that genistein exposure could elevate fetal testicular antioxidative enzyme activity when cotreated with MEHP.

#### 3.2.3 Testosterone assay

**FIGURE 7:**
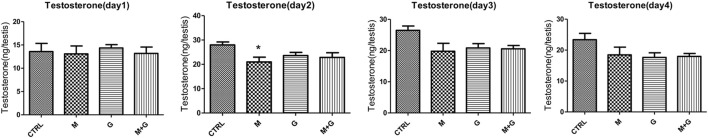
Testosterone concentration analysis. *, significantly different from CTRL at *p* < 0.05.

Testosterone concentration in the culture medium were shown in Figure 7. After cultured for 1 day, no significant alterations were observed in all treated groups (*p* > 0.05). On day two, MEHP exposure induced significant decrease in testosterone concentration in comparison with control (*p <* 0.05). On day three and day four, no significantly decrease of testosterone concentration was found compared with control (*p >* 0.05).

#### 3.2.4 Testicular morphology

**FIGURE 8:**
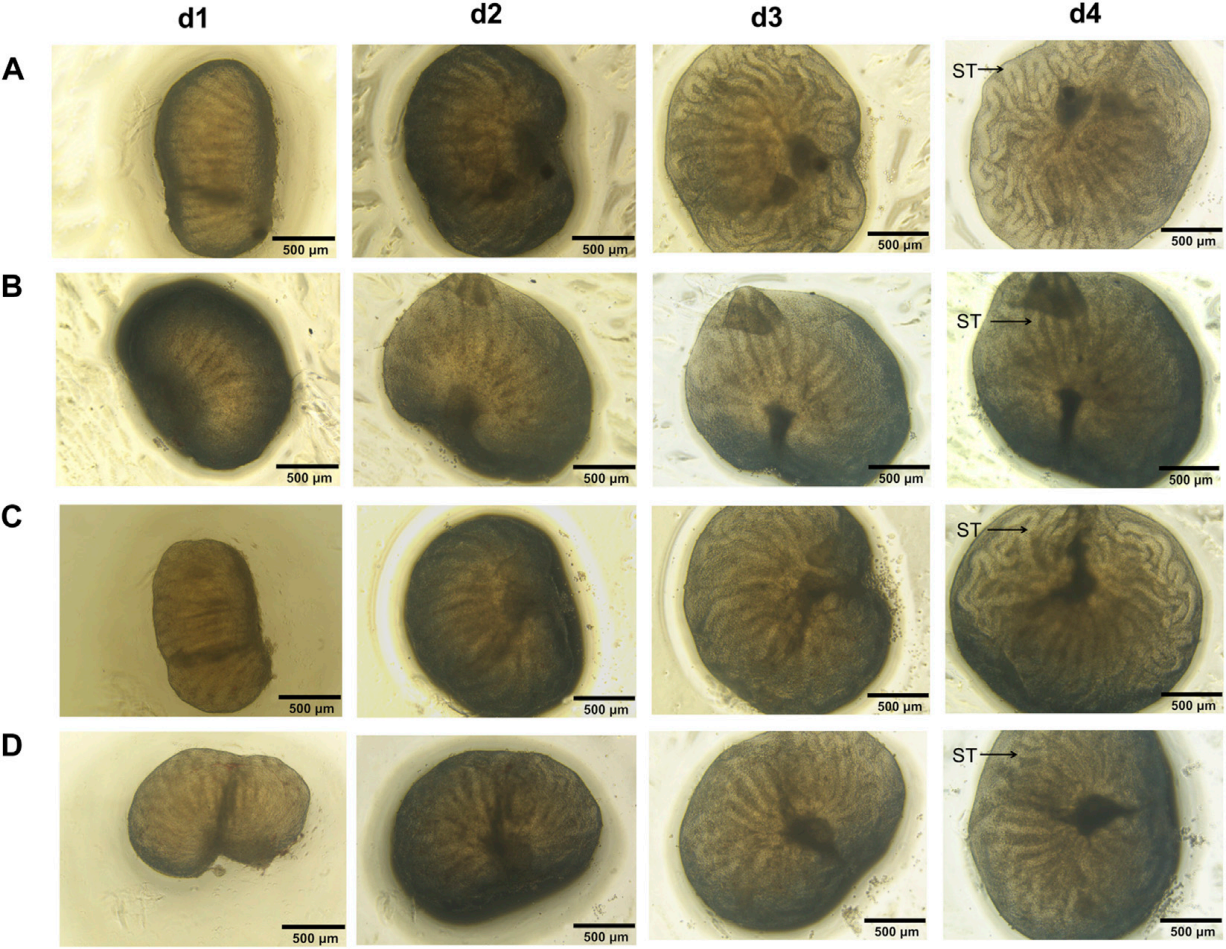
Testicular morphology of the cultured fetal testis. The volume of fetal testis increased, seminiferous tubules on d4 in all groups were more coiled than before and no obvious alteration of testicular morphology was observed in all treated groups. ST, seminiferous tubules. ×40 magnification. Scale bars indicate 500 µm. **(A)** control; **(B)** group M; **(C)** group G; **(D)** group M + G.

When fetal testes were observed under inverted microscope, the volume of fetal testis, the length and thickness of each seminiferous tubule in all groups progressively increased (Figure 8). Seminiferous tubules on d4 in all groups were more coiled and no obvious alterations of testicular morphology were observed in all treated groups, indicating that fetal testis development was maintained *ex vivo* and low dose of MEHP, genistein and their mixture showed no apparent toxic effects on testicular development.

#### 3.2.5 Testicular histology

**FIGURE 9:**
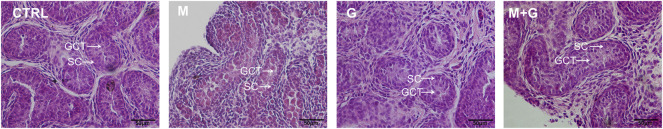
H&E staining of fetal testis cultured for 4 days. H&E staining showed normal testicular structure in all treated groups and tubular lumens was still not formed. GCT, gonocytes; SC, Sertoli cells. ×200 magnification. Scale bars indicate 50 µm.

On d4, intact seminiferous tubules structure was all well sustained in all groups and no obvious necrosis or tubular vacuole was observed. Moreover, no obvious tubular lumens was formed in all groups (Figure 9). Gonocytes were round and located in the center of the tubules, which can readily be distinguished from the Sertoli cells, indicating that low dose of EDCs exposure showed no apparent toxic effects on fetal testicular cell development.

#### 3.2.6 Ultrastructural analysis

**FIGURE 10:**
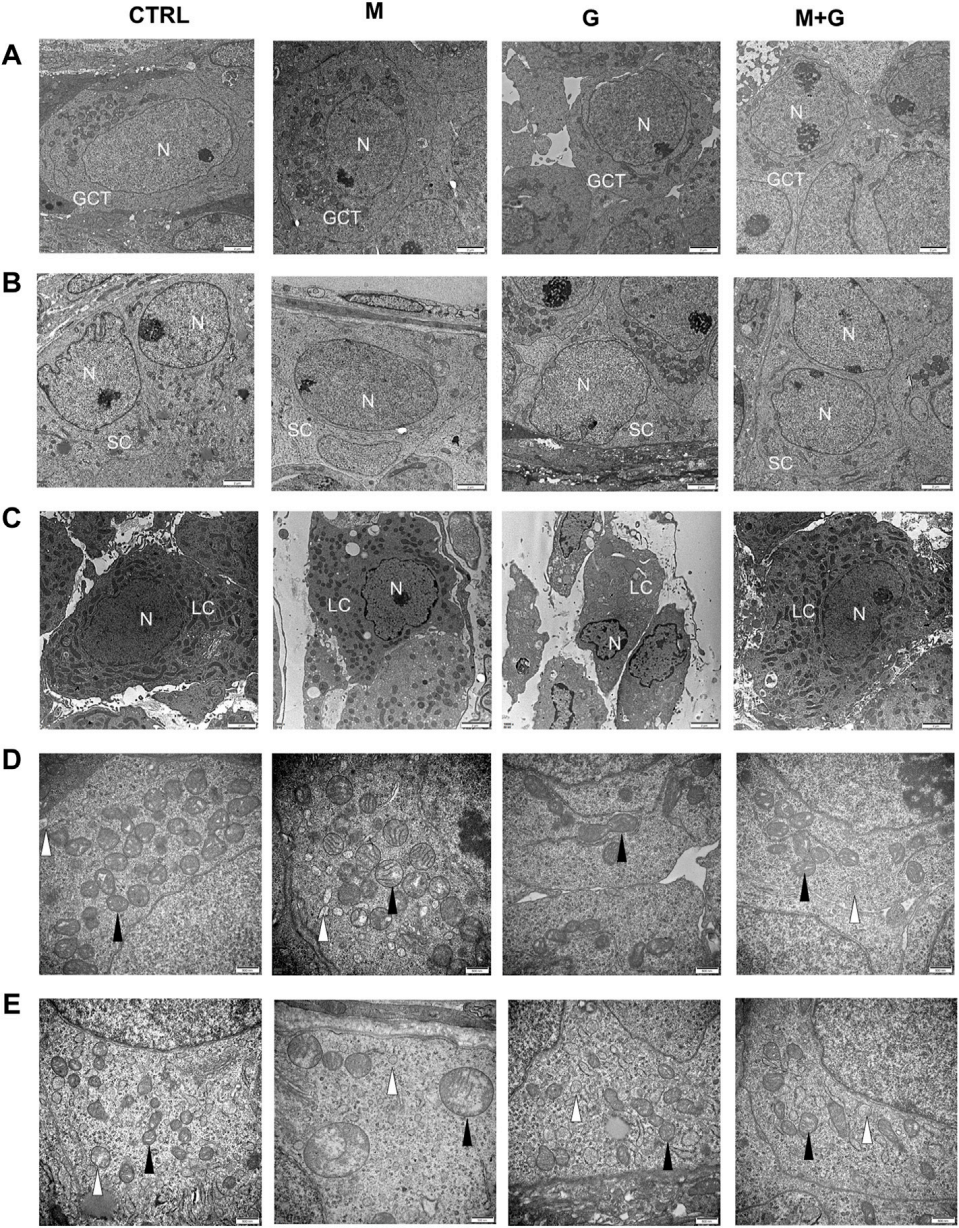
Ultrastructural analysis of the cultured testis. Mitochondria swelling in the gonocytes and Sertoli cells was found after MEHP exposure for 4 days. By contrast, gonocytes, and Sertoli cell in control, group G and M + G exhibited normal ultrastructure. Leydig cells in all groups exhibited normal ultrastructure and intact basement membrane were sustained. GCT, gonocytes; SC, Sertoli cells; LC, Leydig cells; N, nuclei; ▲, mitochondria; ∆, endoplasmic reticulum. **(A)** gonocytes; **(B)** Sertoli cells; **(C)** Leydig cells; **(D)** mitochondria of gonocytes; **(E)** mitochondria of Sertoli cells. **(A–C)** ×10,000 magnification, scale bars indicate 2 μm; **(D,E)** ×30,000 magnification, scale bars indicate 500 nm.

The ultrastructure of gonocytes, Sertoli cells, and Leydig cells was investigated by electron microscopic observations (Figure 10). Mitochondria swelling in the gonocytes was found after MEHP exposure for 4 days, and some of the mitochondrial cristae disappeared. Similar mitochondria lesion was also observed in Sertoli cells after MEHP exposure. By contrast, gonocytes and Sertoli cells in control, group G and M + G exhibited normal ultrastructure, in which endoplasmic reticulum and mitochondria were well defined, and no obvious swelling was found. Intact basement membrane was sustained in all groups. Leydig cells in all groups exhibited normal ultrastructure, mitochondria, and endoplasmic reticulums swelling were also not observed.

## 4 Discussion

Fetal period is critical for testis development and is known as a period of high sensitivity to EDCs (Cargnelutti et al., 2021). During the stage, both functions of testis (spermatogenesis and steroidogenesis) are set up (Culty, 2013). During fetal life, Sertoli cells begin to differentiate from dpc 13.5 to dpc 14.5 and testosterone was synthesized by fetal Leydig cells around 15.5 dpc (Habert and Picon, 1984). Gonocytes surrounded by Sertoli cells begin dividing at 13.5 dpc and enter quiescent phase until postnatal day 2-3 (Manku and Culty, 2015). It is widely accepted that testis development impairment in fetus may lead to testicular dysgenesis syndrome (TDS), which is comprised of poor semen quantity, cryptorchidism, hypospadias and testicular cancer. Further studies demonstrated that exposure to EDCs contributed to the occurance of TDS due to disrupted Leydig cell and Sertoli cell function during fetal life (Perobelli et al., 2012; Erkekoglu et al., 2014).

It was found that some of the EDCs may exhibit “low-dose effects.” The “low-dose effects” was firstly defined by the National Toxicology Program panel of the United States as any biological changes 1) occurring in the range of typical human exposures or 2) occurring at doses lower than those typically used in standard testing protocols, i.e., doses below those tested in traditional toxicology assessments (Vandenberg et al., 2012). Previous studies mostly investigated the toxic effects of single chemical exposure at high doses, however, how multiple EDCs exposure at low doses would affect fetal testis development were rarely studied (Perobelli, 2014). Several *in vitro* approaches were developed to analyze the direct testicular effects of EDCs, however, poor survivability and impaired testis differentiation in those approaches limited their application (van Dissel-Emiliani et al., 1993). Current organ culture system showed obvious advantages by maintaining intact testicular architecture and intercellular communications, which further facilitated *ex vivo* fetal testis development. However, further modification was need to make the culture system more reliable and effective. To achieve this goal, we cultured testis of 15.5 dpc fetal rat and assessed whether testis development was better sustained using modified agarose organ culture system. Delayed seminiferous tubules development and smaller tubules were observed in the control. By contrast, we found that after cultured for 4 days, the fetal rat testis showed increased volume, elongated and coiled seminiferous tubules, indicating fetal testis development was sustained *ex vivo* and the agarose organ culture system could better mimic normal physiological process. Seminiferous tubules formation and elongation were mainly based on the proliferation of Sertoli cells. It was found that Leydig cells could stimulate the proliferation of Sertoli cells by secreting Activin A (Yoshida, 2010). However, the molecular mechanisms involved in tubular growth and elongation remain to be determined. It was reported that the elongation and coiling of testis tubules occurred between E16.5 and E19.5 in rats (Nakata et al., 2021). As a result of Sertoli cell proliferation, the testis tubules elongate and become coiled to increase the Sertoli cell surface and maximally use the available testis volume, which is probably an indication that the intratesticular space becomes limiting due to emergence of the tunica albuginea (Makela et al., 2019). So in this process, tubule elongation is accompanied by tubule coiling, and the appearance of more coiled tubules in the modified group means the modified system is more suitable for fetal testis development. In this regard, the modified culture system may serve as a tool for further study to elucidate the mechanism involved in tubules formation. Furthermore, testosterone concentration of the culture medium showed increase in the first 3 days and the modified group showed higher testosterone concentration compared with control. The initiation of testosterone production in fetal rat starts at 15.5 dpc and reaches a peak at 18.5 dpc (Habert and Picon, 1984), which was in accordance with *ex vivo* findings in this study, further indicating that the modified culture system could provide proper environment for fetal rat testis development and mimic the physiological process *in vivo*.

Based on the modified agarose culture system, we further found that exposure to low dose of MEHP, genistein and their mixture at 0.1 μmol/L, induced mild developmental disruption of fetal germ cells, and no obvious alterations was found in Leydig cell and Sertoli cell markers. It was supposed that DEHP and genistein may exert low-dose effects at doses equivalent to human exposures or below those used for traditional toxicological studies (Vandenberg et al., 2012). One recent *in vivo* research by Culty M revealed that gestational exposure (from gestational day 14 to birth) to a mixture of GEN and DEHP at 0.1 mg/kg/day, increased the rates of infertility and abnormal testicular morphology in adult rats, manifesting that the non-monotonic effects of low dose of EDCs in fetal life may contribute to the reproductive effects in adult (Walker et al., 2020). In fetal testis, gonocytes arise from primordial germ cells (PGCs) and give rise to spermatogonial stem cells (SSCs) (Culty, 2013). In rat testis, gonocytes begin to divide from 13.5 dpc and gradually migrate to the basement membrane of the seminiferous tubules from the center of the testicular cords postnatally (Manku and Culty, 2015). In our study, the gonocyte marker Sohlh2 was downregulated after single and combined exposure to genistein and MEHP. Among all regulators, Sohlh2 is normally expressed in pre-meiotic germ cells and function as an important spermatogenesis-specific transcription factor (Toyoda et al., 2009). It was found that Sohlh2-deficient testis looked normal at birth but exhibited reduced spermatogonia number by postnatal day seven. Sohlh2 deficiency were also identified to disrupts normal spermatogenesis by regulating Gfra1, Sox3, and Kit gene expression (Park et al., 2016). It was reasonable to speculate that *in utero* accumulation of EDCs in fetus and immature metabolic systems of fetus may contribute to more apparent testicular injuries or typical “low-dose effects” than *ex vivo* study at low concentration. The mechanism of low-dose effects may attribute to indirect effects of EDCs by displacing endogenous steroids from the reservoir on plasma binding sites, especially when EDCs showed strong competition for steroid binding plasma proteins. Moreover, the circulating EDCs in majority maintained physiologically active in the body, thus the EDCs would be expected to disturb normal physical function and induce obvious pathological alterations at low doses (Milligan et al., 1998).

Among all cell types in the fetal testis, Leydig cells was firstly affected after MEHP exposure at low doses, followed by decreased gonocytes proliferation and disrupted Sertoli cell function (Chauvigne et al., 2009). Testosterone production showed significant decrease after MEHP exposure at 0.1 μmol/L only on day two, while the testosterone concentration on the other 3 days seemed to be recovered. Physically fetal testis starts to produce testosterone at dpc 15.5 and gradually reaches a peak at dpc 18.5 in the rat (Johnson et al., 2012). Similar trend was also observed in this study, showing testosterone concentration increase after cultured for 2 and 3 days. Previous study reported that Sertoli cells appeared vacuolated and anti-Müllerian hormone (AMH) production decreased after MEHP exposure at the dose of 10 μmol/L in an organ culture system (Chauvigne et al., 2009), moreover, testosterone concentration was reduced to the threshold of detection (Chauvigne et al., 2009). MEHP exposure at 1 μmol/L showed no obvious changes of AMH but induced decreased testosterone concentration, exhibiting antiandrogenic effects (Chauvigne et al., 2009). However, the toxic effects for doses equivalent to 0.1 μmol/L or even lower were scarcely tested. It is also interesting to note that coexposure to genistein and MEHP at 0.1 μmol/L revealed no significant alteration in testosterone concentration during organ culture, manifesting that the combined effects of EDCs mixture exposure may be greatly different from individual outcomes. Furthermore, it is also reasonable to speculate that although there were multiple evidences supporting the low-dose effects, exposure to genistein, MEHP, and their mixture at the dose of 0.1 μmol/L did not provide strong evidence to this theory.

In physiological state, testis has an endogenous antioxidant system which protects it against oxidative damage. Normally ROS regulate a wide range of cellular functions and intracellular antioxidant systems could cope with ROS damage, however, excessive ROS can induce cellular reduction-oxidation (redox) imbalance, followed by successive pathophysiological alterations. It was found that phthalates activated peroxisome proliferator-activated receptors (PPARs) and suppressed testosterone biosynthesis (Culty et al., 2008), in this process testicular redox state imbalance and excessive ROS production contributed to the toxicological injuries. Our previous studies observed that high doses of MEHP exposure aggravated Sertoli cell apoptosis, accompanied by decreased testicular antioxidative enzyme activities and elevated intracellular ROS production (Zhang et al., 2017; Zhang et al., 2018). Ultrastructurally, MEHP exposure at 0.1 μmol/L induced apparent mitochondria swelling and part of the mitochondrial cristae disappeared inside the gonocytes, while genistein and the mixture group showed no obvious ultrastructure alterations. In accordance with ultrastructure findings, we also found that SOD activity in the medium decreased significantly after MEHP exposure. Genistein was referred to as an estrogen chemical compound present in some plants, more recent studies revealed that it may modulate cellular antioxidative ability (Yarmohammadi et al., 2021). Our previous *in vivo* study found that exposure to DEHP *in utero* at low dose disturbed the redox balance at PND3, while coexposure to genistein alleviated DEHP induced cellular injuries, suggesting a protective role of genistein after short-term DEHP exposure (Jones et al., 2015). It is reasonable to speculate that EDCs mixture may act not simply in the manner of adding or subtracting of individual chemical (Silins and Hogberg, 2011; Vandenberg et al., 2012) and the combined effects may differ greatly from single chemical exposure.

## 5 Conclusion

In this study, we modified agarose culture system and evaluated the testicular effects after MEHP and genistein exposure at low dose. We found that the modified agarose culture system could better mimic testicular microenvironment without obvious hypoxic cell damage, and the development of fetal somatic and germ cells was well sustained. Our results also revealed that MEHP at 0.1 μmol/L exerted mild disruption to fetal testis development by altering gonocyte development, inducing mitochondrial injuries and transient reduction of testosterone production. However, genistein at 0.1 μmol/L could partially protect fetal testis against MEHP-induced damage by balancing testicular redox state. It is reasonable to speculate that genistein at low dose may exhibit curative effects on fetal testis by attenuating plasticizer induced reproductive injuries during early life.

## Data Availability

The original contributions presented in the study are included in the article/supplementary materials, further inquiries can be directed to the corresponding author.
